# Chaperone-E3 Ligase Complex HSP70-CHIP Mediates Ubiquitination of Ribosomal Protein S3

**DOI:** 10.3390/ijms19092723

**Published:** 2018-09-12

**Authors:** Inwoo Hwang, Sung-Woo Cho, Jee-Yin Ahn

**Affiliations:** 1 Department of Molecular Cell Biology, Sungkyunkwan University School of Medicine, Suwon 16419, Korea; inwhwang@skku.edu; 2 Single Cell Network Research Center, Sungkyunkwan University School of Medicine, Suwon 16419, Korea; 3 Department of Biochemistry and Molecular Biology, University of Ulsan, College of Medicine, Seoul 05505, Korea; swcho@amc.seoul.kr; 4 Samsung Biomedical Research Institute, Samsung Medical Center, Seoul 06351, Korea

**Keywords:** neuronal apoptosis, ribosomal protein S3 (RPS3), Akt/PKB (protein kinase B), carboxy terminus of heat shock protein 70-interacting protein (CHIP)

## Abstract

In addition to its role in ribosome biogenesis, ribosomal protein S3 (RPS3), a component of the 40S ribosomal subunit, has been suggested to possess several extraribosomal functions, including an apoptotic function. In this study, we demonstrated that in the mouse brain, the protein levels of RPS3 were altered by the degree of nutritional starvation and correlated with neuronal apoptosis. After endurable short-term starvation, the apoptotic function of RPS3 was suppressed by Akt activation and Akt-mediated T70 phosphorylation, whereas after prolonged starvation, the protein levels of RPS3 notably increased, and abundant neuronal death occurred. These events coincided with ubiquitination and subsequent degradation of RPS3, controlled by HSP70 and the cochaperone E3 ligase: carboxy terminus of heat shock protein 70-interacting protein (CHIP). Thus, our study points to an extraribosomal role of RPS3 in balancing neuronal survival or death depending on the degree of starvation through CHIP-mediated polyubiquitination and degradation.

## 1. Introduction

Ribosomal protein S3 (RPS3) is a component of the 40S ribosomal subunit and is involved in ribosome maturation [1]. Increasing evidence suggests that ribosomal proteins have extraribosomal functions. The most extensively documented extraribosomal function in many types of cancer cells is the inhibition of a p53 negative regulator called mouse double minute homologue 2 (MDM2) by RPL5 and RPL11 [2,3]. As other important extraribosomal functions, RPS3 possesses a general base damage endonuclease activity that participates in the cleavage of DNA lesions caused by UV irradiation and other types of DNA-damaging agents [4,5]. Both RPS3 and ribosomal protein P0 have apurinic/apyrimidinic (AP) endonuclease activity that is implicated in DNA repair functions on the 3′ side of AP sites after DNA damage [4,5,6]. In addition, ribosomal proteins are involved in apoptosis as follows: RPS3a is thought to participate in the apoptosis of NIH3T3 cells [7], and a knockdown of RPS3 leads to improved cell survival after H_2_O_2_ treatment [8]. Moreover, in breast cancer cells, a knockdown of RPS3 induces apoptosis by downregulating X-linked inhibitor of apoptosis (XIAP) [9]. Nonetheless, RPS3 has previously been reported to induce antiapoptotic genes such as X-linked inhibitor of apoptosis by activating nuclear factor κB [10]. Recent studies by Russo and colleagues have also revealed that in the absence of p53, overexpression of RPL3 in cancer cells induces cell cycle arrest or apoptosis by upregulating p21 [11], and in the presence of the chemotherapeutic drug fluorouracil (5-FU), RPL3 triggers mitochondrial apoptosis through the overproduction of Bax and inhibition of Bcl2 as well as the prevention of nuclear translocation of NF-κB via the upregulation of IκB [12,13]. Additionally, we have previously shown that overexpression of RPS3 causes neuronal apoptosis with an upregulation of proapoptotic proteins Dp5 (i.e., Hrk) and Bim, and this apoptotic function is largely diminished by Akt-mediated T70 phosphorylation, leading to nuclear accumulation of RPS3 [14] after stimulation by a growth factor. Nevertheless, under physiological conditions, the extraribosomal role of RPS3 as an apoptotic inducer remains unclear.

It has been reported that the cellular function of RPS3 is regulated by post-transcriptional modifications such as phosphorylation, sumoylation, and ubiquitination. For instance, T42 phosphorylation by Erk1 and S6/T221 phosphorylation by protein kinase C-δ [15,16] are required for nuclear translocation and the DNA repair function of RPS3, respectively. T70 phosphorylation by Akt blocks the proapoptotic function of RPS3 [14]. Sumoylation of RPS3 or an interaction with heat shock protein 90 (Hsp90) results in resistance to proteasome-dependent degradation, thus enhancing protein stability [17,18]. Ubiquitination of RPS3 by Asc1 (RACK1 in mammals) is a critical event for ribosome-associated quality control [19]. Additionally, pVHL, the product of the von Hippel–Lindau (VHL) tumor suppressor gene, functions as the substrate recognition component of an E3-ubiquitin ligase complex targeting hypoxia-inducible factor α for ubiquitination and degradation and associates with RPS3 but does not target it for degradation [20]. Either overexpression or knockdown of RPS3 expression can lead to apoptosis, suggesting that the total abundance of RPS3 is crucial for cell survival [8,14,21]. However, the underlying mechanisms governing the regulation of RPS3 expression under specific environmental conditions remain unclear.

Neuronal cell death is induced by many stressors, including starvation, which causes apoptosis and plays fundamental roles in brain development and neurological diseases [22,23]. It is well-known that the phosphatidylinositol 3-kinase (PI3K)–Akt cascade is a key pathway responsible for the prosurvival effects of neurons under various conditions [24,25,26], and the activation of PI3K–Akt signaling protects neurons from starvation-induced apoptosis [27,28]. Previously, we have shown that RPS3 is an Akt substrate involved in neuronal survival and can act as a potent molecular switch that can regulate apoptotic induction and DNA repair in neurons [14]. In this study, we demonstrated that during nutrient starvation in the mouse brain, the protein levels of RPS3, but not its mRNA levels, prominently increased and contributed to neuronal apoptosis after severe prolonged starvation (PS). In contrast, after relatively short-term starvation (STS), RPS3 was downregulated while Akt signaling was activated, followed by RPS3 phosphorylation. These protein levels appeared to be regulated by the ubiquitin–proteasome system (UPS). Moreover, we found that HSP70 and cochaperone E3 ligase, the carboxy terminus of heat shock protein 70 (HSP70)-interacting protein (CHIP), bound directly to RPS3 and ubiquitinated it for proteasomal degradation. These findings indicate that RPS3 is a substrate for CHIP and revealed the molecular mechanism by which neuronal cells coordinate survival and apoptosis under starvation conditions.

## 2. Results

### 2.1. RPS3 Expression Is Highly Upregulated in the Hippocampus and Cortex of the Brain upon Starvation

Neuronal apoptosis can be induced by starvation and increased protein levels of RPS3. To investigate the role of RPS3 in starvation-induced apoptosis in the brain, mice were subjected to starvation for different periods, and the mouse brain hippocampus or cortex was isolated.

**Figure 1:**
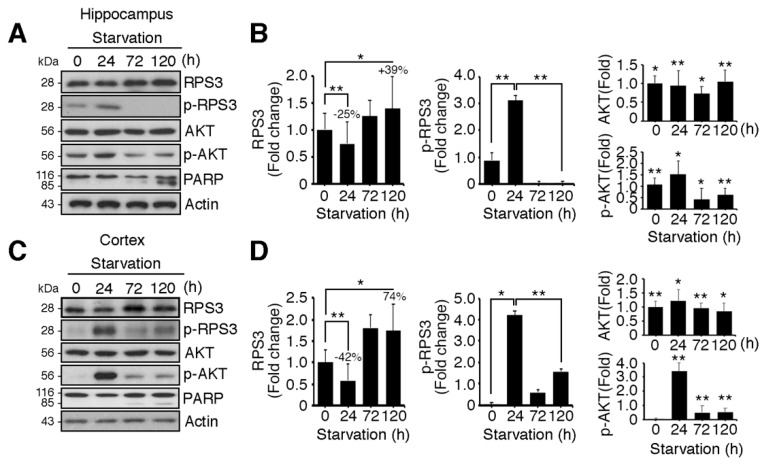
Ribosomal protein S3 (RPS3) is highly upregulated in the hippocampus and cerebral cortex after starvation. C57BL/6 mice were subjected to nutrient starvation, with only water supplied, for 0, 24, 72, and 120 h and were euthanized to isolate the hippocampal area and cortical area of the brains. Endogenous protein levels of RPS3 or phospho (p)-RPS3 in the (**A**) hippocampus or (**C**) cortex were measured by immunoblotting with anti-RPS3 and anti-p-RPS3 antibodies, and neuronal apoptosis was evaluated with an anti-poly-ADP-ribose polymerase (PARP) antibody. (**B**,**D**) RPS3/p-RPS3 (left), AKT/p-AKT (right) protein levels were determined by densitometric analysis in (**B**) the hippocampus or (**D**) cortex. Densities of immunoblotting were measured with Image J and its normalized with control mice (0 h) brain lysates. Values in this figure represent the mean ± SEM from three independent experiments, and the images shown are representative of at least three independent experiments. Statistical significance was calculated using a one-way ANOVA test followed by Turkey’s post-test (* *p* < 0.01; ** *p* < 0.001).

We determined both the mRNA levels and protein levels of RPS3 in the hippocampus and cerebral cortex of starved mice. Of note, we found that the protein levels of RPS3 were slightly decreased after relatively STS (24 h), whereas PS enhanced the expression of RPS3 proteins in both the hippocampus and cortex (Figure 1A–D). Nonetheless, no significant changes in RPS3 mRNA levels were observed during starvation (Figure S1A,B). Akt was activated after starvation (24 h) as determined by quantification of phosphorylated Akt, and its phosphorylation notably decreased as starvation continued (72–120 h) (Figure 1A,C, fourth panels, and Figure 1B,D, middle graphs). These results are in line with those of previous studies showing that Akt is activated by various stressful conditions for survival when the damage is not yet lethal, but when insults are prolonged, Akt activation is decreased or abrogated [29,30]. In agreement with our previous study [14], phospho-RPS3 levels correlated with activated Akt (Figure 1A,C, second panels, Figure 1B,D, left graphs). Moreover, when Akt activation was terminated showing diminished phosphorylation of Akt and RPS3 expression increased (after 72 h), the cleavage of poly-ADP-ribose polymerase (PARP), a well-known marker of apoptosis, increased after continued starvation in comparison with the absence of starvation or STS conditions (24 h). These data indicated that apoptosis increased after PS (at 72 and 120 h; Figure 1A,C, fifth panels, Figure 1B,D, right-hand graphs). We have previously demonstrated that under DNA damage conditions, RPS3 is phosphorylated at threonine (T) 70 by Akt and therefore cannot induce neuronal apoptosis. However, RPS3 elicits profound apoptotic death after prolonged genotoxic stress, when RPS3 fails to be phosphorylated by Akt as Akt activation is terminated [14]. Similarly, our data suggest that under STS conditions, RPS3 is phosphorylated by Akt activation and delayed neuronal death, whereas when starvation persists, the expression of RPS3 is highly elevated but its phosphorylation is abrogated as Akt activation declines.

### 2.2. Starvation Induced Neuronal Death by Upregulating RPS3 and Decreasing Phosphorylation of RPS3-T70

**Figure 2:**
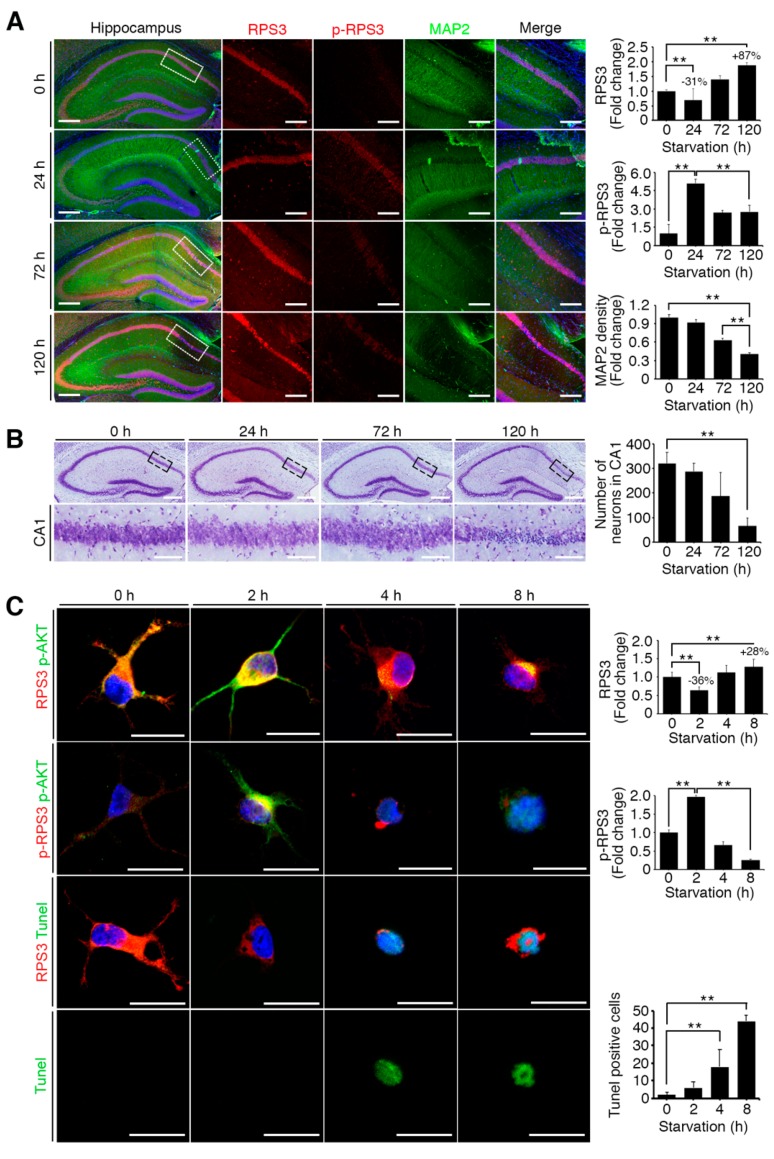

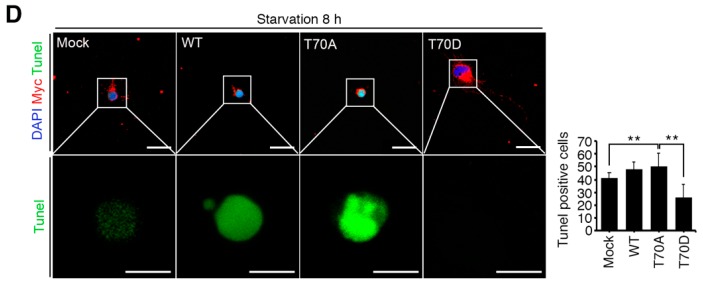
Starvation induced neuronal death by upregulating RPS3 and decreasing phosphorylation of RPS3-T70. (**A**) Brains of starved mice were isolated and subjected to immunohistochemical analysis. The hippocampus was stained for RPS3 (red, left) or p-RPS3 (red, right) and MAP2 (green). The right-hand panel shows higher magnification of the CA1 region of the hippocampus, indicated by a white box. Scale bar, 1 mm (left), 100 μm (right). Fluorescence intensities were measured by ZEN (ZEISS) and their values were normalized with normal mouse brain. (**B**) Neuronal cells in CA1 were stained with DAPI (blue) and an anti-NeuN antibody (green), and apoptotic cells with condensed-chromatin signs were counted. Arrows indicate death of cells. Scale bar, 1 mm (upper), 100 μm (bottom). (**C**) A representative merged image of protein expression levels of endogenous RPS3 or p-RPS3 (red) and p-AKT (green) with or without B27 serum starvation condition (short-term starvation, STS, or prolonged starvation, PS) in the hippocampal neurons (upper). Apoptotic cells quantified with the TUNEL assay (green) and an anti-RPS3 antibody (red, left), and TUNEL-positive cell numbers are presented in the bar graph (right). Scale bar, 20 μm. (**D**) Cultured neurons were transfected with Myc-RPS3 WT, T70A, T70D, or Myc control vector on day 1 after plating (DIV 1) and incubated with or without B27 at DIV 3. The cells were fixed and co-stained with an anti-Myc antibody (red) and subjected to a TUNEL assay (green). The mice (*n* = 20) were subdivided into four groups: Normal and Starvation for 24, 72, and 120 h. Quantification of neuronal cell survival rates from three independent experiments is shown (right). Scale bar, 20 μm; Statistical significance was calculated using a one-way ANOVA test followed by Turkey’s post-test (* *p* < 0.01; ** *p* < 0.001).

To verify whether starvation induces neuronal apoptosis in response to the induction of RPS3 expression and reduction of its T70 phosphorylation, we subjected mice to STS (24 h) and PS (72 and 120 h) and isolated the brains for immunohistochemical analysis. The results showed that RPS3 expression decreased after 24 h starvation but increased after an additional 72 and 120 h of starvation in the hippocampus, whereas the amount of T70-phospho RPS3 was elevated at 24 h and diminished after 72 h, accompanied by decreased MAP2 intensity (Figure 2A). Moreover, quantification of neuronal apoptosis by DAPI staining suggested that NeuN-positive neurons underwent apoptotic death and displayed condensed chromatin after 72 h of starvation (Figure 2B). These data suggest that STS induced Akt-mediated RPS3 T70 phosphorylation and slightly decreased RPS3 protein levels, as well as attenuated neuronal apoptosis, whereas PS promoted neuronal loss by increasing RPS3 protein levels and decreasing RPS T70 phosphorylation. 

We next detected neuronal apoptosis in primary cultured hippocampus neurons. Compared to control neurons without starvation, approximately 10% of neurons were TUNEL-positive at 2 h (STS condition for primary cultured neurons). However, TUNEL-positive cells greatly increased in number to more than 40% and 75% at 4h and at 8 h of starvation (PS condition for primary cultured neurons), respectively, in response to increased RPS3 levels, suggesting that PS augmented the protein levels of RPS3, leading to neuronal death (Figure 2C). Additionally, neurons expressing RPS3 WT or T70A (a nonphosphorylatable mutant) underwent apoptosis after prolonged starvation, whereas overexpression of T70D (phosphorylation-mimetic mutant) resulted in neuronal survival with normal morphology even under PS conditions (Figure 2D), indicating that T70 phosphorylation of RPS3 attenuates the proapoptotic function of RPS3.

### 2.3. RPS3 Is Regulated by UPS under Starvation Conditions

**Figure 3:**
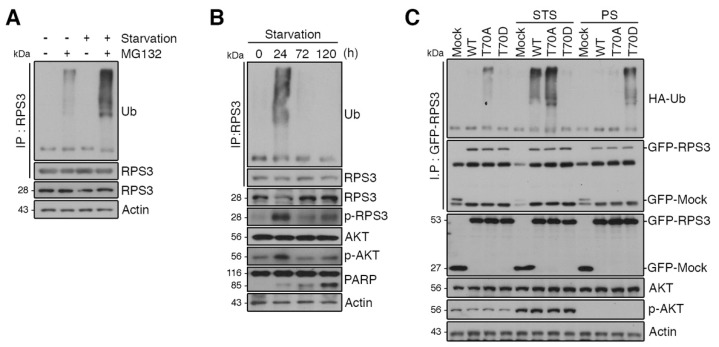
RPS3 is regulated by the ubiquitin–proteasome system (UPS) under starvation conditions. (**A**) Cortical neurons were incubated with or without the proteasome inhibitor MG132 and/or B27 serum starvation for 2 h. The cell lysates were subjected to immunoprecipitation with the anti-RPS3 antibody. Ubiquitinated RPS3 levels were detected by immunoblotting with an anti-Ub antibody. (**B**) Brain lysates of starved mice (0, 24, 72, and 120 h) were subjected to immunoprecipitation with the anti-RPS3 antibody. Ubiquitination of endogenous RPS3 was measured by immunoblotting with the anti-Ub antibody. (**C**) GFP-RPS3 WT, T70A (a nonphosphorylatable mutant), T70D (a phosphorylation-mimetic mutant), or control vector were transfected into HEK 293T cells together with HA-Ub and incubated with or without FBS for STS (8 h) or PS (16 h). Starvation conditions for these HEK293 cells were determined by immunoblotting (Figure S2). Immunoprecipitation of the cell lysates was performed with an anti-GFP antibody to detect ubiquitination of transfected RPS3 WT, T70A, or T70D with an anti-HA antibody.

The protein levels of RPS3 decreased after STS and increased after PS, but the mRNA levels of RPS did not change. Thus, we wondered whether RPS3 protein expression is regulated by UPS-dependent degradation under starvation conditions. Pretreatment with MG132, a proteasomal inhibitor, efficiently accumulated polyubiquitinated RPS3, and starvation increased RPS3 polyubiquitination in HEK293 cells (Figure 3A), indicating that starvation conditions regulate RPS3 protein amounts by promoting ubiquitination-dependent proteasomal degradation. To test whether RPS3 ubiquitination is related to RPS3 protein levels under STS or PS conditions, we monitored the ubiquitination of RPS3 under various starvation conditions using mouse brain lysates. Indeed, RPS3 polyubiquitination increased after STS and then decreased after PS. These results indicated that RPS3 stability is UPS-dependent during starvation (Figure 3B).

To determine whether the stability of RPS3 during starvation is regulated by ubiquitination, we transfected HEK293 cells with GFP-tagged RPS3 WT, T70A, or T70D constructs. In normal media, ubiquitination was slightly increased in RPS WT or T70A compared to in the T70D mutant, while RPS3 WT or T70A ubiquitination was markedly increased upon starvation (STS) and T70D remained resistant to ubiquitination, indicating that Akt-mediated T70D phosphorylation interrupts RPS3 ubiquitination. Of note, when starvation persisted, RPS3 WT or T70A ubiquitination decreased, whereas T70D ubiquitination increased, suggesting that RPS3 protein levels decreased during STS and increased after PS in the mouse brain (Figure 3C).

### 2.4. RPS3 Is a Novel Substrate of a Chaperone-E3 Ligase Complex HSP70/CHIP for Degradation

**Figure 4:**
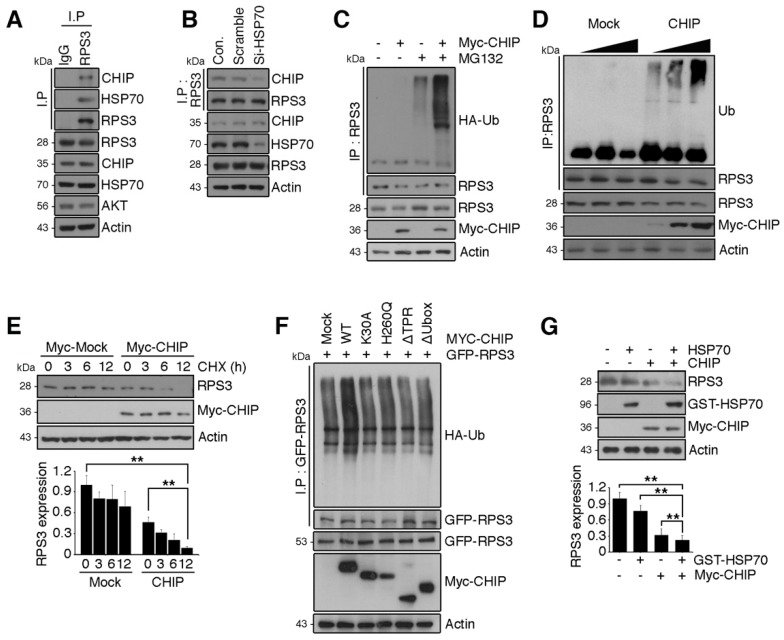
RPS3 is a novel substrate of a chaperone-E3 ligase complex heat shock protein 70 (HSP70)–CHIP (carboxy terminus of HSP70-interacting protein) for degradation. (**A**) HEK 293T cell protein lysates were subjected to immunoprecipitation with an anti-IgG or anti-RPS3 antibody and subjected to immunoblotting with anti-CHIP, HSP70, or Akt antibodies. (**B**) Lysates of 293T cells transfected with si-RNA scrambled or si-HSP70 were subjected to immunoprecipitation with the anti-RPS3 antibody and immunoblotting with an anti-CHIP antibody. Endogenous HSP70 protein levels were analyzed by means of the anti-HSP70 antibody. (**C**) Cortical neurons were transfected with Myc-tagged CHIP or its control vector, and the cells were incubated with or without the proteasome inhibitor MG132. The neurons were lysed and processed for immunoprecipitation with the anti-RPS3 antibody. Ubiquitination levels of endogenous RPS3 were measured by immunoblotting with the anti-HA antibody. Immunoprecipitation was performed to detect ubiquitinated RPS3. (**D**) 293T cells were transfected with various amounts of Myc-CHIP (2/4 μg), and the cell lysates were subjected to immunoprecipitation with the anti-RPS3 antibody. Ubiquitinated RPS3 was detected by immunoblotting. (**E**) Transfected 293T cells were treated with cycloheximide (CHX, 10 μM) at the indicated time points (0, 3, 6, or 12 h) and probed during the immunoblotting with an anti-RPS3, anti-Myc, or anti-actin antibody (upper panel). Quantification of the RPS3 protein levels by densitometry analysis (bottom). (**F**) Cells were co-transfected with Myc-CHIP WT or various mutant versions: ΔTPR, K30A, ΔUbox, or H260Q, as well as GFP-RPS3. The transfected cells were incubated with MG132. Ubiquitinated RPS3 was quantified by means of the anti-HA antibody. (**G**) Myc-CHIP was co-transfected with GST-HSP70 into 293T cells, and protein levels of RPS3 were detected with the anti-RPS3 antibody (top). Quantification of the RPS3 protein levels by densitometric analysis (bottom). Densities of immunoblotting were measured with Image J and their values were normalized with normal cell lysate or control. Statistical significance was calculated using a one-way ANOVA test followed by Turkey’s post-test (* *p* < 0.01; ** *p* < 0.001). Values in this figure represent mean ± SEM from three independent experiments, and the image shown is representative of at least three independent experiments.

Previous studies showed that monoubiquitination of RPS3 by Asc1 (RACK1 in mammals) or Hel2 is required for ribosome-associated quality control [19,31] but does not cause proteasomal degradation of RPS3. Although treatment with the HSP90 inhibitor elicited UPS-dependent degradation of RPS3, recruiting HSP70 [18], the identity of the E3 ligase responsible for RPS3 degradation and its biological significance have not been reported. Because the molecular chaperone HSP70 is recruited to RPS3 in the presence of HSP90, we tested whether HSP70 and cochaperone E3 ligase, CHIP, which directs chaperone substrates for ubiquitination and proteasomal degradation, are involved in RPS3 degradation. We found that RPS3 forms a complex with endogenous HSP70 and CHIP (Figure 4A), and this RPS3–HSP70 interaction is a prerequisite for trimeric-complex formation. Depletion of HSP70 prevented the association between RPS3 and CHIP (Figure 4B).

We next determined whether CHIP functions as an E3 ligase for RPS3 degradation. Overexpression of CHIP promoted RPS3 ubiquitination, and RPS3 ubiquitination was increased in a dose-dependent manner by CHIP expression levels (Figure 4C,D), indicating that CHIP is a ubiquitin ligase for RPS3 protein degradation. Moreover, the half-life of RPS3 was markedly decreased in CHIP-expressing cells compared to control cells (Figure 4E). Furthermore, the deletion mutation of the TPR domain in CHIP or K30A mutation, which strongly affects the association with HSP70, was less effective for RPS3 ubiquitination, showing levels similar to those yielded by the control vector. Additionally, a deletion mutant of Ubox or the H260Q mutant form with no E3 ligase activity of CHIP also produced much lower ubiquitination of RPS3 compared to WT-CHIP (Figure 4F). Accordingly, cotransfection of CHIP with HSP70 notably diminished RPS3 protein levels (Figure 4G), demonstrating that RPS3 is an E3 ligase substrate of CHIP, and HSP70 is necessary for CHIP-mediated RPS3 degradation. 

### 2.5. RPS3 Degradation Is Regulated by HSP70/CHIP under Starvation Conditions

**Figure 5:**
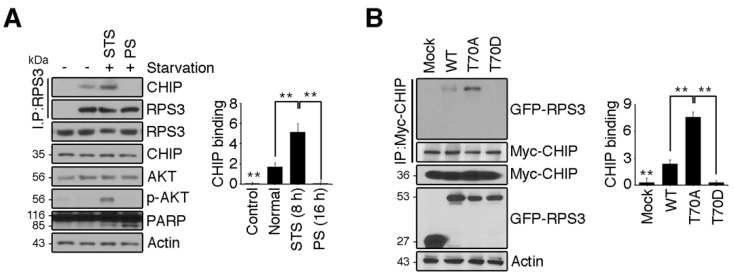
RPS3 degradation is regulated by HSP70/CHIP under starvation conditions. (**A**) HEK 293T cells were incubated with or without FBS. Endogenous RPS3 and CHIP binding affinities were determined by means of anti-RPS3 and anti-CHIP antibodies after immunoprecipitation with the anti-RPS3 antibody. (**B**) GFP-RPS3 WT, T70A, T70D, or control vector were transfected into HEK 293T cells with Myc-CHIP. Protein lysates were subjected to immunoprecipitation with the anti-Myc antibody, and binding affinities with RPS3 mutant forms were determined by immunoblotting with an anti-GFP antibody. Western blot intensities were measured by Image J and their values were normalized with normal cell lysate (A) or mock transfected cell (B). Values in this figure represent the mean ± SEM from three independent experiments, and the images shown are representative of at least three independent experiments. Statistical significance was determined using a one-way ANOVA test followed by Turkey’s post-test (* *p* < 0.01; ** *p* < 0.001).

Because starvation-mediated neuron apoptosis occurs due to high levels of RPS3 expression and the abrogation of T70 phosphorylation, we hypothesized that CHIP regulates RPS3 degradation in response to starvation. To test this idea, we determined whether the association of CHIP and RPS3 was altered during starvation. Consistent with our finding that RPS3 ubiquitination was noticeably elevated during STS and attenuated during PS (Figure 3B), the interaction between CHIP and RPS3 was strong during STS (8 h) and significantly decreased during PS (16 h; Figure 5A). These data indicated that CHIP promoted RPS3 ubiquitination and subsequent degradation under starvation conditions but prevented the dissociation of ubiquitin from RPS3 as starvation continued. Our binding analysis showed that CHIP strongly interacted with the RPS3 T70A mutant, but only minimally interacted with the WT and showed no detectable interaction with the T70D mutant, suggesting that T70 phosphorylation prevents CHIP binding (Figure 5B). Presumably, during STS, RPS3 is degraded by binding to CHIP, whereas phospho-T70 RPS3, which suppresses its own proapoptotic function, escapes from CHIP-mediated degradation. After PS, RPS3 is highly expressed and induces apoptosis, along with dissociating from CHIP, and phospho-RPS3 is downregulated after Akt inactivation. 

## 3. Discussion

**Figure 6:**
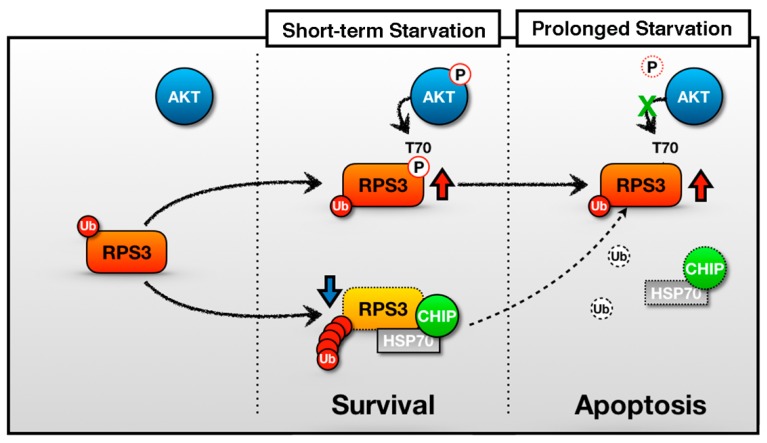
A schematic of the representative role of RPS3 in STS and PS. In the short-term starvation condition, RPS3 T70 is phosphorylated by p-Akt and its phosphorylated form induces protein stability. Moreover, RPS3 (the nonphosphorylated form) is regulated by UPS-dependent degradation with a chaperone-E3 ligase complex, HSP70–CHIP, leading to neuronal cell survival. Otherwise, after prolonged starvation, the interaction of the RPS3 and HSP/CHIP complex is significantly reduced, and its ubiquitination is decreased. RPS3 is highly expressed and induces neuronal apoptosis. Red arrow = protein stability is increased; blue arrow and dotted line = protein stability is decreased.

The decision for a neuron to protect itself from death or to switch on its death program may be affected by the magnitude of stress. Previously, we have proposed an extraribosomal function of RPS3 as an apoptotic inducer regulated by Akt-mediated T70 phosphorylation [14]. A model for RPS3-induced neuronal apoptosis at different degrees of starvation is proposed here (Figure 6). In this study, we showed that if stressors are not lethal (STS is not lethal), neurons activate their survival signals (e.g., Akt and subsequent RPS3 phosphorylation). If the stress is too severe or persists (e.g., PS examined in this study), then a neuron can activate intrinsic apoptotic pathways, leading to the accumulation of RPS3 (which may serve as a potent inducer of apoptosis) through a release from its E3 ligase, CHIP.

Ribosomal proteins are active not only in protein translation but also in multiple extraribosomal processes including DNA repair, tumorigenesis, and apoptotic cell death [32]. As an important extraribosomal function in many types of cancer cells, it has been suggested that ribosomal proteins play roles in cell cycle progression and apoptosis either p53-dependently or p53-independently in response to nucleolar stress because the nucleolus is implicated in the sensing of (and responses to) cellular stress by stabilizing p53 [13]. Under nucleolar stress, several ribosomal proteins (e.g., RPL11, RPL5, RPS6, and RPS7) relocate to the nucleoplasm, where they can bind to MDM2, thus enhancing p53 stability and subsequent p53-dependent cell cycle arrest and apoptosis [2,3,33,34,35]. On the other hand, RPL3 acts as a stress-sensing molecule in response to drug-induced stress in cancer cells lacking p53. RPL3 elicits G1 phase arrest through transcriptional induction of p21, a cyclin-dependent kinase inhibitor, and induces apoptosis in p53-deficient cells under nucleolar stress induced by actinomycin D [36]. Likewise, RPL3 accelerates apoptotic death during a loss of p53 function by inhibiting NF-κB nuclear translocation via stabilization of the IκB-a protein after 5-FU treatment, which can induce nucleolar stress [37]. The stress-sensing ability of the nucleolus has been also demonstrated in neurons [38,39], and nucleolar stress is involved in the pathogenesis of neurodegenerative diseases including Huntington’s disease, Parkinson’s disease, and Alzheimer’s disease [40]. We found that in neuronal cells, RPS3 can be translocated to the nucleus upon genotoxic stresses [14] and acts as a critical regulator of apoptotic death after starvation. The extraribosomal function of brain cell nucleoli beyond their involvement in the p53 stress response has not been investigated. Therefore, it will be worthwhile to explore neuroprotective roles such as the proapoptotic and/or antiapoptotic function(s) of ribosomal proteins including RPS3 in response to brain damage.

Although autophagy has long been believed to culminate in neuronal death during starvation [41], accumulating evidence suggests that apoptosis is also an important cell death mechanism in neurons under starvation conditions. For instance, the removal of the serum or nutrients from cultures delays neuronal death, and apoptotic features such as nuclear condensation and caspase 3 activation are observed [42]. Serum deprivation in Neuro-2A cells and hippocampal neurons elicits caspase 9 and 3-associated apoptosis, but not caspase 8 and 12 cleavage [43]. Moreover, under amino acid starvation conditions, alleviation of autophagy by Beclin-1 depletion elevates apoptotic death of hippocampal neurons [44]. Additionally, serum starvation promotes epithelial cell apoptosis induced by Bcl2/Bax [45], and goat skin fibroblast apoptosis is enhanced 3- to 10-fold after 48 and 120 h of serum starvation [46]. Given that neuronal death often occurs in hostile environments such as disease, infection, traumatic, ischemic, and starvation conditions, understanding of the neuroprotection mechanisms for these apoptotic stimuli is necessary for the development of therapeutic approaches.

Our results suggest that during PS, severe neuronal death occurs in the brain (Figure 1 and Figure 2), and that stabilization of the RPS3 protein conferred by its dissociation from its novel E3 ligase partner CHIP contributes to neuronal apoptosis (Figure 5A). Additionally, our data showed that the T70D phosphorylation-mimetic mutant form of RPS3 only minimally interacted with CHIP, whereas the T70A nonphosphorylatable mutant bound to CHIP more tightly (Figure 5B). Based on the nuclear accumulation of phospho-RPS3 or T70D mutant form and upregulation of its endonuclease activity [14] as well as predominant cytoplasmic localization of HSP70–CHIP [47], the nuclear retention of RPS3 by Akt-mediated T70 phosphorylation may have delayed its degradation and allowed RPS3 to act as an endonuclease for DNA repair after PS. Nevertheless, decreased Akt activation was coupled to the loss of RPS3 phosphorylation, and in turn the apoptotic function of RPS3 was provoked under PS conditions, enabling escape from UPS. In addition to Akt-dependent phosphorylation, other posttranslational modifications of RPS3 may occur during starvation that regulate the association of RPS3 and HSP70–CHIP, because WT or T70A ubiquitination decreased while T70D ubiquitination increased during PS (Figure 3C). Presumably during PS, specific post-translational modifications occur in the WT or T70A that ablate the access of CHIP–HSP70. It is also possible that prolonged starvation triggers nuclear translocation of the CHIP–HSP70 complex, because we observed that transfected T70D RPS3, which predominantly resides in the nucleus, is greatly ubiquitinated during PS. Although it is well-established how phosphorylation is important for ubiquitination by E3 ligases and subsequent proteolysis, the regulation of spatial and/or temporal phosphorylation-dependent ubiquitination is understudied. Besides, to what extent phosphorylation can positively or negatively regulate substrate recognition needs further exploration, and further studies are needed to determine how the interaction between RPS3 and HSP70–CHIP is regulated depending on the degree of starvation.

CHIP is a U-box-dependent E3 ligase and possesses three tandem tetra-tri-copeptide repeat motifs that provide a binding interface for the chaperones HSP70 and 90. In this way, CHIP ubiquitinates chaperone-bound substrates [48,49]. Many oncoproteins, including receptor tyrosine kinase ErbB2, hypoxia-inducible factor 1α, and the p85 regulatory subunit of PI3K have been reported to be targets of CHIP. We found that RPS3 is a substrate of CHIP and that the interaction between CHIP and RPS3 appears to be regulated by cellular stress during starvation. Further studies are necessary to determine whether CHIP-mediated RPS3 degradation helps to prevent neuronal death in starvation and whether manipulation of RPS3 stability by CHIP provides novel insights for the development of novel therapeutics for neurological diseases.

## 4. Materials and Methods

### 4.1. Primary Neurons and Cell Culture

The hippocampus of E18 rat pups was isolated into a 15 mL tube containing 10 mL of Hank’s Balanced Salt Solution on ice. Trypsin/EDTA was added to digest the tissue. The samples were incubated for 20 min at 37 °C. Digestion was stopped by washing the hippocampi twice with 4 mL of a complete medium (containing 10% fetal bovine serum (FBS)). Next, 3 mL of the Neuro basal medium (NB, Invitrogen, Carlsbad, CA, USA, 21103-049)/B27 (Invitrogen 17504-044) was added, and the tissue was dissociated by gently triturating the hippocampi through a fire-polished Pasteur pipette. The cell mixture was diluted to 10 mL with NB/B27 and then filtered through a 40 or 70 μm strainer. The cells were centrifuged at 1500× *g* for 5 min and resuspended in 10 mL of NB/B27. HEK293T cells were cultured in Dulbecco’s modified Eagle’s medium supplemented with 10% of FBS and 100 U/mL penicillin and streptomycin in a humidified incubator at 37 °C with 5% CO_2_.

### 4.2. Antibodies, siRNA, and Chemicals

Polyclonal anti-RPS3- and anti-phospho-RPS3 Thr-70-specific antibodies were generated by injecting purified His-tagged RPS3 or synthesized Thr-70-phosphorylated peptide (CLGEKGR-RIRELpTAV) into rabbits. Anti-Akt, anti-p-Akt, anti-CHIP, anti-PARP, and anti-caspase-9 antibodies were acquired from Cell Signaling Technology (Danvers, MA, USA). Anti-actin, anti-Myc, anti-HA, anti-GST, and anti-GFP antibodies were purchased from Santa Cruz Biotechnology (Dallas, TX, USA). Anti-HSP70 was bought from Abcam (Cambridge, UK). The Alexa Fluor 594-conjugated goat anti-rabbit IgG antibody and Alexa Fluor 488-conjugated goat anti-mouse IgG antibodies (secondary antibodies) were acquired from Molecular Probes (Eugene, OR, USA). The siRNA for Si-HSP70 (5′-GAAGGACGAGUUUGAGCACAA-3′) was bought from Cosmogentech (Seoul, Republic of Korea). Cycloheximide was purchased from Duchefa Biochemie (Haarlem, The Netherlands), and MG132 from Sigma (St. Louis, MO, USA). Lipofectamine 3000 was acquired from Invitrogen.

### 4.3. Construction of Recombinant DNA

RPS3 was cloned into the pcDNA3.1 Myc-His and pEGFP-C2 vectors. PCR-based mutagenesis was performed with the QuikChange site-directed mutagenesis kit (Stratagene, La Jolla, CA, USA) and using the following primers: T70A forward, 5′-CGGATTCGGGAACTGGCAGCTGTAGTTCAGAAG-3′, and reverse, 5′-CTTCTGAACTACAGCTGCCAGTTCCCGAATCCG-3′) and T70D forward, 5′-CGGATTCGGGAACTGGACGCTGTAGTTCAGAAG-3′, and reverse, 5′-CTTCTGAACTACAGCGTCCAGTTCCCGAATCCG-3′).

### 4.4. Mouse Starvation

This study was reviewed and approved by the Institutional Animal Care and Use Committee (IACUC) of Sungkyunkwan University School of Medicine (SUSM) (code SKKUIACUC-17-6-3-1/SKKUIACUC-17-6-5-1, approval date 25 April 2017). SUSM is an Association for Assessment and Accreditation of Laboratory Animal Care International (No. 001004) accredited facility, and abides by Institute of Laboratory Animal Resources guidelines. All experimental procedures were carried out in accordance with the regulations of the IACUC guideline of Sungkyunkwan University. Six- to eight-week-old C57BL/6 male mice (Orient Bio, Seongnam, Korea) were singly housed in cages. Food was removed for up to 5 days, but the mice were allowed free access to water. Room temperature was 23 °C, and the light cycle was 12 h light and 12 h dark. The mice were monitored following by guideline, measuring their weight (Figure S3) and general health condition (Table S1) every day. The mice (*n* = 20) were subdivided into four groups: Normal and Starvation for 24, 72, and 120 h. Data on neuronal cell death are expressed as means of triplicate measurements from three independent experiments. 

### 4.5. Western Blotting

Transfected cells were washed with PBS and treated with ice-cold lysis buffer (50 mM Tris-Cl, pH 7.4, 150 mM NaCl, 1 mM EDTA, 0.5% Triton X-100, 1.5 mM Na_3_VO_4_, 50 mM sodium fluoride, 10 mM sodium pyrophosphate, 10 mM β-glycerolphosphate, 1 mM phenylmethylsulfonyl fluoride, and protease cocktail (Calbiochem, San Diego, CA, USA)). The cell extracts were clarified by centrifugation at 20,000× *g* for 10 min. Proteins were denatured and resolved by SDS-PAGE and transferred to nitrocellulose membranes (Pall Life Science, Port Washington, NY, USA). The membranes were blocked in 5% skim milk and incubated sequentially with primary antibodies and horseradish peroxidase-conjugated secondary antibodies. All of original image for western blotting were provided as Figures S4 and S5.

### 4.6. Coimmunoprecipitation Assay and In Vitro Binding Assay

Cells were rinsed with phosphate-buffered saline (PBS) and lysed in a buffer (50 mM Tris-Cl, pH 7.4, 150 mM NaCl, 1 mM EDTA, 0.5% Triton X-100, 1.5 mM Na_3_VO_4_, 50 mM sodium fluoride, 10 mM sodium pyrophosphate, 10 mM β-glycerolphosphate, 1 mM phenylmethylsulfonyl fluoride, and protease cocktail (Calbiochem)). Cell lysates (0.5–1 mg of protein) were mixed with a primary antibody with protein A/G beads and incubated for 3 h at 4 °C with gentle agitation. The beads were then washed in lysis buffer, mixed with 2× SDS sample buffer, boiled, and analyzed by immunoblotting.

### 4.7. Ubiquitination Assay

HEK 293T cells were cotransfected with GFP or Myc-tagged RPS3 WT, T70A, or T70D and Myc-CHIP and HA-Ub, and then incubated in a humidified incubator at 37 °C with 5% CO_2_ for 24 h. Cells were treated with MG132 10 μM (Sigma-Aldrich, St. Louis, MO, USA). The cells were washed with PBS and lysed in ice-cold lysis buffer for 30 min. The cell extracts were clarified by refrigerated centrifugation at 20,000× *g* for 10 min, and the lysates (1 mg) were mixed with anti-GFP or Myc antibodies with protein A/G beads and incubated for 3 h at 4 °C with gentle agitation. The beads were then centrifuged at 1000× *g* for 1 min and washed three times using ice-cold lysis buffer. The immunoprecipitated proteins remaining on the beads were boiled in 2× SDS sample buffer for 10 min, resolved by 8% SDS-PAGE, and transferred onto a nitrocellulose membrane. Anti-HA antibody for HA-Ub and anti-GFP or Myc antibodies for GFP or Myc-RPS3 WT, T70A, or T70D were used to evaluate the ubiquitination of RPS3.

### 4.8. Immunofluorescence

Hippocampal neurons were isolated from E18.5 and plated on a coverslip coated with poly-D-lysine (25 mg/mL). The neurons were transfected with a Myc-tagged RPS3 WT, T70A, T70D, or control vector using Lipofectamine 3000 (Invitrogen) at DIV 1 (day after plating). The neurons were incubated with or without B27 serum for 8 h at DIV 3. The neurons were fixed for 30 min at room temperature with 4% paraformaldehyde in PBS. Those cells were incubated overnight at 4 °C with a primary antibody, washed with ice-cold PBS, and then incubated for 1 h at room temperature with secondary antibody, Alexa Fluor 488 goat anti-mouse (Invitrogen). Nuclei were counterstained with DAPI. Immunostained images were acquired by means of a laser scanning confocal microscope (LSM 710, Carl Zeiss, Oberkochen, Germany). The confocal microscope was controlled by the ZEN software.

### 4.9. TUNEL Assay

The TUNEL staining was conducted using a commercial kit, DeadEnd Fluorimetric TUNEL system from Promega (Madison, WI, USA). In brief, neurons were pretreated with 20 μg/mL proteinase K for 5 min and then incubated with the reaction mixture containing terminal deoxynucleotidyl transferase (TdT) and fluorescein-conjugated deoxyuridine triphosphate (dUTP) for 1 h at 37 °C. Cells were immunostained using primary anti-Myc antibody and the appropriate Alexa Fluor 594 goat anti-mouse secondary antibody. Nuclei were co-interstained with DAPI stain. Cells with condensed nuclei or positivity for TUNEL staining were counted as positive (green) for cell death with transfected positive signal (red). More than 50 random cells were counted for each individual sample. Immunostained images were acquired using a laser scanning confocal microscope (LSM 710, Carl Zeiss). The confocal microscope was controlled using ZEN software and the acquisition was performed in the Research Core Facility.

### 4.10. Immunohistochemistry

Mouse brains were removed from the euthanized mice, fixed in 4% paraformaldehyde, and incubated with 30% sucrose in PBS for 48 h. The brains were frozen in OCT compound and cut coronally into 8-μm-thick slices. The sections were washed with PBS and permeabilized with 0.25% Triton X-100 in PBS for 10 min, and blocked in 1% bovine serum albumin for 30 min. The cells were immunostained with primary antibodies (anti-RPS3, p-RPS3, and anti-MAP2 or anti-NeuN) overnight at 4 °C. Stained tissues were mounted with a mounting medium (Vector Laboratories, Burlingame, CA, USA).

### 4.11. Statistical Analysis

Densitometry analysis of immunoblotting used Image J software and the values were normalized by actin. Immunohistochemistry or immunocytochemistry images were analyzed by ZEN software (ZEISS, Oberkochen, Germany). Data are presented as mean ± S.D. of three independent experiments. Statistical analysis was performed with Jamovi software using one-way analysis of variance (ANOVA). Differences with *p* < 0.05 were considered statistically significant (** *p* < 0.001; * *p* < 0.01); n.s., no statistically significant difference (*p* ≥ 0.05).

## Supplementary Materials

Supplementary materials can be found at http://www.mdpi.com/1422-0067/19/9/2723/s1.
