# Supplementary material for: Chaperone-E3 Ligase Complex HSP70-CHIP Mediates Ubiquitination of Ribosomal Protein S3
Source: Int J Mol Sci. 2018 Sep 12;19(9):2723. doi: 10.3390/ijms19092723 (PMC6163665; doi:10.3390/ijms19092723)
Supplement: Supplementary file 1 [file ijms-19-02723-s001.pdf]

International Journal of
Molecular Sciences

MDPI

Article

Chaperone-E3 ligase complex HSP70/CHIP mediates ubiquitination of ribosomal protein S3

Inwoo Hwang [1,2], Sung-Woo Cho [4] and Jee-Yin Ahn [1,2,3]*

[1] Department of Molecular Cell Biology, Sungkyunkwan University School of Medicine, Suwon 16419, Korea; inwhwang@skku.edu (I.H.); jeeahn@skku.edu (J.Y.A.)
[2] Single Cell Network Research Center, Sungkyunkwan University School of Medicine, Suwon 16419, Korea; inwhwang@skku.edu (I.H.); jeeahn@skku.edu (J.Y.A.)
[3] Samsung Biomedical Research Institute, Samsung Medical Center, Seoul, 06351 Korea; jeeahn@skku.edu (J.Y.A.)
[4] Department of Biochemistry and Molecular Biology, University of Ulsan, College of Medicine, Seoul 05505, Korea; swcho@amc.seoul.kr (S.W.C.)
* Correspondence: jeeahn@skku.edu; Tel.: +82-31-299-6134; Fax: +82-31-299-6139

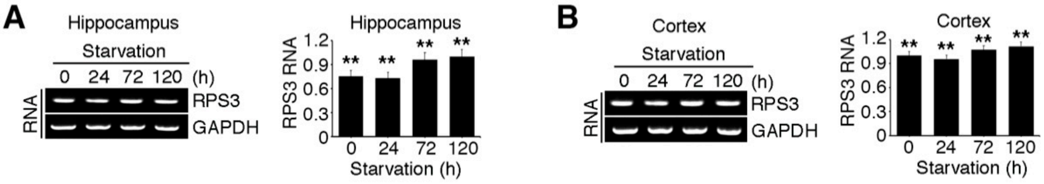

Figure S1. RNA expression levels of RPS3 are no significant changed from starved brains. C57BL/6 were subjected to nutrient starvation. The RNA levels of RPS3 were measured in the (A) hippocampus or (b) cortex by RT-PCR. The expressions were determined by densitometric analysis and its values were normalized with its normal brains (0 h). Statistical significance was calculated using a one-way ANOVA test followed by Turkey's post-test **P < 0.001). Values in this figure represent the mean ± SEM from three independent experiments and the image shown is representative of at least three independent experiments.

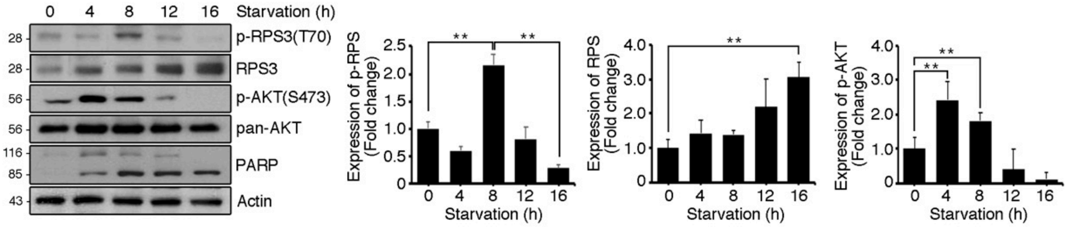

Figure S2. Endogenous expression levels of RPS3, p-RPS3, AKT, p-AKT or PARP were measured for time interval 0, 4, 8, 12 or 16 hr after FBS serum starvation in HEK 293T cells by immunoblotting with indicating antibodies. Protein expression of p-RPS3 (left), RPS3 (middle) and p-AKT (right) were determined by densitometric analysis and its values were normalized with actin. Statistical significance was calculated using a one-way ANOVA test followed by Turkey's post-test (**P < 0.001). Values in this figure represent the mean ± SEM from three independent experiments, and the image shown is representative of at least three independent experiments.

A

Descriptives

Starvation time	N	Mean	SD
0	15	25.7	1.234
24	15	24.4	1.298
48	15	22.1	1.580
72	15	20.7	1.175
120	11	18.2	0.751

B

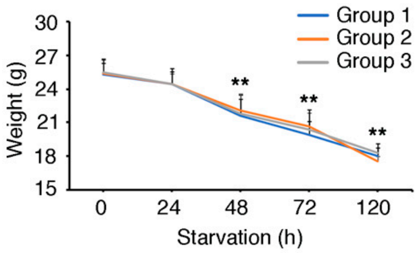

C

Post Hoc Comparisons - Starvation time

Comparison							
Starvation time		Starvation time	Mean Difference	SE	df	t	ptukey
0	-	24	1.27	0.461	62.0	2.74	0.059
	-	48	3.60	0.461	62.0	7.80	< .001
	-	72	4.92	0.489	62.0	10.05	< .001
	-	120	7.57	0.516	62.0	14.67	< .001
24	-	48	2.33	0.461	62.0	5.06	< .001
	-	72	3.65	0.489	62.0	7.46	< .001
	-	120	6.30	0.516	62.0	12.21	< .001
48	-	72	1.32	0.489	62.0	2.69	0.067
	-	120	3.97	0.516	62.0	7.69	< .001
72	-	120	2.65	0.541	62.0	4.90	< .001

Figure S3. Six- to eight-week old C57BL/6 male mice (Orient Bio) were fasting treatment. The mice (total number=60) were subdivided into 4 groups with starvation time, observing their weight and general health condition. During fasting treatment, 4 mice were sacrificed by following ethics guide line. (A) The average weights of mice were calculated by grouping starvation time using a one-way ANOVA test and (B) the graph was showed its weight curve. (C) Body weights were calculated using one-way ANOVA test followed by Turkey's post-test (**P < 0.001).

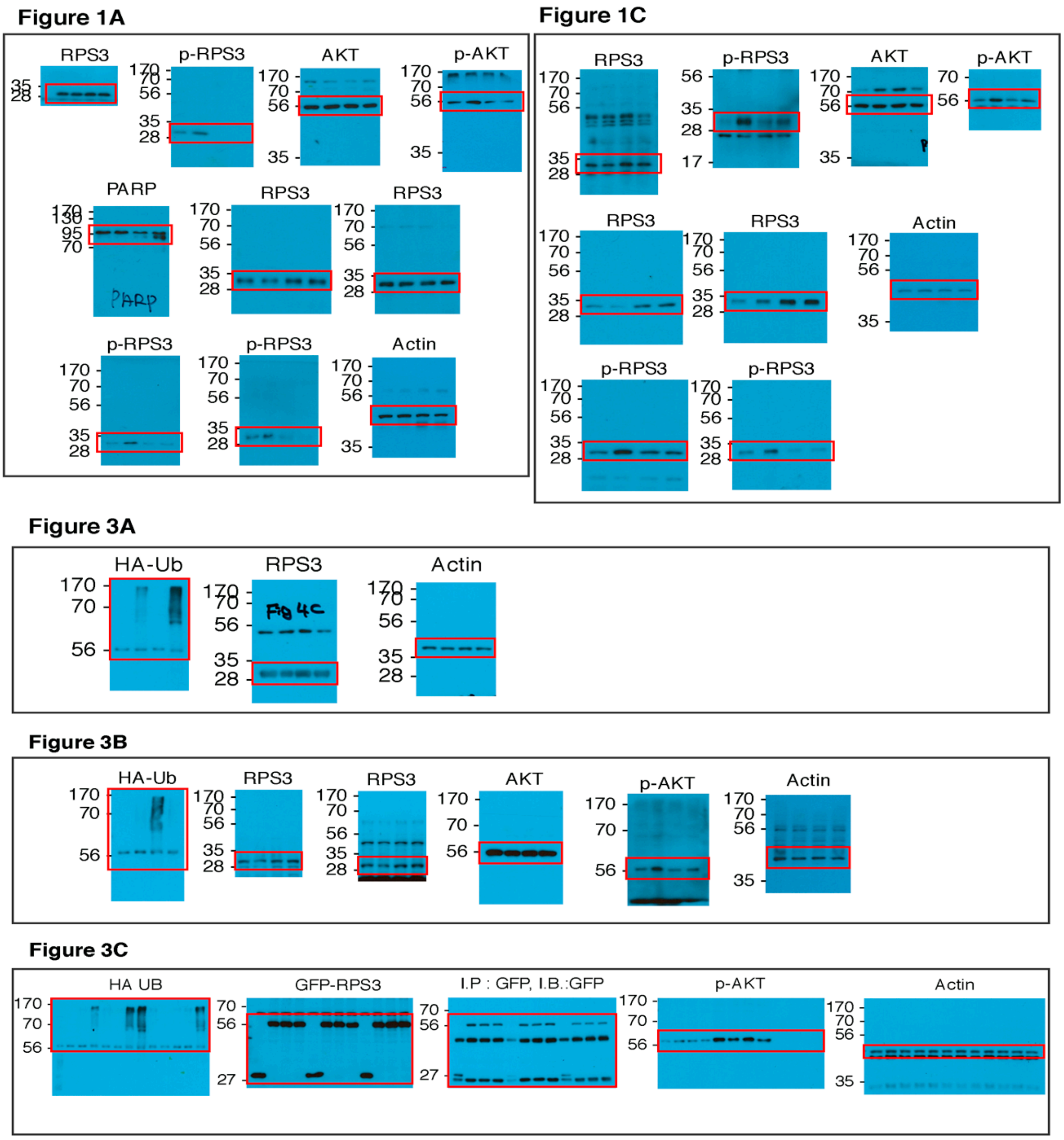

Figure S4. Unprocessed original scans of Western blot analysis in the indicated figures. Scanned film image of Figure 1-3 immunoblotting experiment. Labels on top of each membrane scan indicate the antibody used for detection. Dashed red boxes show the regions that were cropped for the figures. Molecular size markers in kDa.

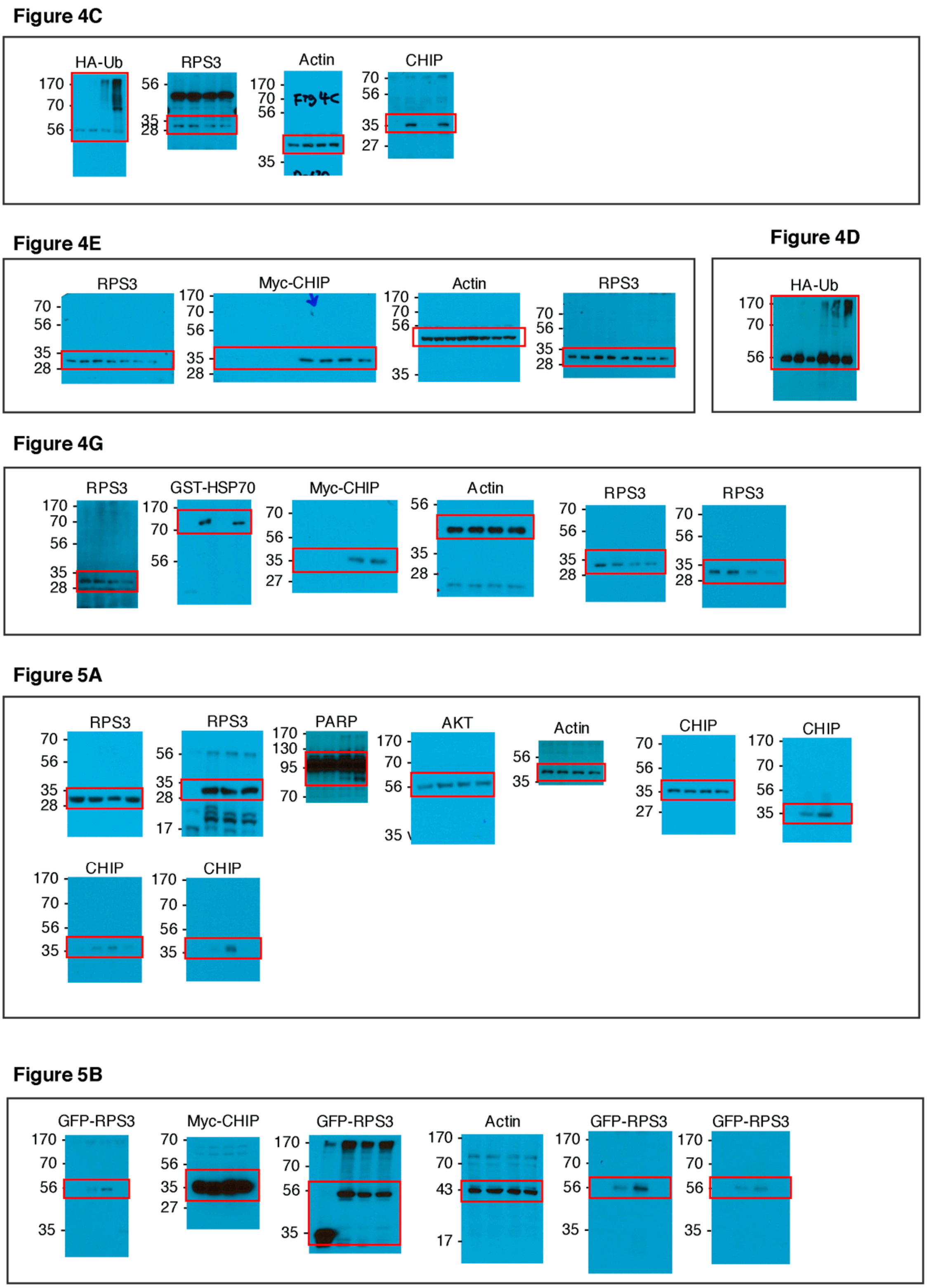

Figure S5. Uncropped immunoblots referring to Figure 4-5. Labels on top of each membrane scan indicate the antibody used for detection. The canned images were cropped around the molecular weight of the respective proteins and were presented in the figures. Dashed red boxes show the regions that were cropped for the figures. Molecular size markers in kDa.

Group 1

		NUMBER	Score	A1-1	A1-2	A1-3	A1-4	A1-5	B1-1	B1-2	B1-3	B1-4	B1-5	C1-1	C1-2	C1-3	C1-4	C1-5	D1-1	D1-2	D1-3	D1-4	D1-5
Scoring sheet Group 1	Weight loss	Normal	0	0	0	0	0	0	0	0	0	0	0										
		10%	1																				
		10~15%	2											2	2	2	2						
		> 20%	3															3		3	3	3	
	Appearance	Normal	0	0	0	0	0	0	0		0		0			0							
		Small change	1							1		1		1	1		1	1		1	1	1	
		Body Temp. 1~2 ℃<br>Heart beats, breath rates +30%	2																				
		Body Temperture > 2 ℃<br>Heart beats, breath rates +50%	3																				
	Body condition	BCS > 3	0	0	0	0	0	0	0	0	0	0	0		0	0	0						
		BCS 2~3	1											1				1		1	1	1	
		BCS 1~2	2																				
		BCS < 1	3																				
			Total score	0	0	0	0	0	0	1	0	1	0	4	3	2	3	5		5	5	5	

Group 2

		NUMBER	Score	A2-1	A2-2	A2-3	A2-4	A2-5	B2-1	B2-2	B2-3	B2-4	B2-5	C2-1	C2-2	C2-3	C2-4	C2-5	D2-1	D2-2	D2-3	D2-4	D2-5
Scoring sheet Group 2	Weight loss	Normal	0	0	0	0	0	0	0	0	0	0	0										
		10%	1											1	1								
		10~15%	2														2	2					
		> 20%	3													3			3	3	3	3	3
	Appearance	Normal	0	0	0	0	0	0	0	0			0	0									
		Small change	1								1	1			1	1	1	1			1	1	
		Body Temp. 1~2 ℃<br>Heart beats, breath rates +30%	2																2				2
		Body Temperture > 2 ℃<br>Heart beats, breath rates +50%	3																				
	Body condition	BCS > 3	0	0	0	0	0	0	0	0	0	0	0	0	0		0						
		BCS 2~3	1													1			1	1	1	1	
		BCS 1~2	2																				2
		BCS < 1	3																				
			Total score	0	0	0	0	0	0	0	1	1	0	1	2	5	3	3	6	4	5	5	7

Group 3

		NUMBER	Score	A3-1	A3-2	A3-3	A3-4	A3-5	B3-1	B3-2	B3-3	B3-4	B3-5	C3-1	C3-2	C3-3	C3-4	C3-5	D3-1	D3-2	D3-3	D3-4	D3-5
Scoring sheet Group 3	Weight loss	Normal	0	0	0	0	0	0	0	0	0	0	0										
		10%	1																				
		10~15%	2											2	2	2		2					
		> 20%	3														3			3	3		3
	Appearance	Normal	0	0	0	0	0	0		0	0	0	0		0								
		Small change	1						1					1		1	1	1		1	1		1
		Body Temp. 1~2 ℃<br>Heart beats, breath rates +30%	2																				
		Body Temperture > 2 ℃<br>Heart beats, breath rates +50%	3																				
	Body condition	BCS > 3	0	0	0	0	0	0	0	0	0	0	0	0	0	0		0					
		BCS 2~3	1																	1			1
		BCS 1~2	2														2				2		
		BCS < 1	3																				
			Total score	0	0	0	0	0	1	0	0	0	0	3	2	3	6	3		5	6		5

Table S1. Mice health monitoring sheet. During fasting treatment, mice were observed weight loss, appearance (small change, body temperature or heart beats) and body condition (BCS rates) every day. Total 60 mice were used for experiments, 20 mice for each experiments and mice (n=4, grey box) were sacrificed, decreasing its body weight more than 20% a day before experimental day.
